# Improved biorefinery pathways of marine diatoms using a water miscible ionic liquid and its colloidal solution: efficient lipid extraction and *in situ* synthesis of fluorescent carbon dots for bio-imaging applications†

**DOI:** 10.1039/d1ra01425k

**Published:** 2021-06-22

**Authors:** Paidi Murali Krishna, Veerababu Polisetti, Krishnaiah Damarla, Subir Kumar Mandal, Arvind Kumar

**Affiliations:** Academy of Scientific and Innovative Research (AcSIR) Ghaziabad 201002 India damarlakittu@gmail.com skmandal@csmcri.res.in arvind@csmcri.res.in; CSIR-Central Salt and Marine Chemicals Research Institute G. B. Marg Bhavnagar 364002 Gujarat India

## Abstract

In this study, a water-miscible ‘classic’ ionic liquid (IL), 1-ethyl-3-methylimidazoliumacetate ([EMIM][Ac]), has been used for lipid extraction from marine diatoms *Thalassiosira lundiana* CSIR-CSMCRI 001 by following a non-polar solvent partition method. The composition of lipid was determined using gas chromatography-mass spectrometry (GC-MS). In total, 91.4 mg g^−1^ (dry wt) of lipid was produced, out of which the percentage of docosahexaenoic acids (DHA), myristic acid, palmitic acid, and arachidonic acid was 19.6%, 15.1%, 11.2%, and 10.4%, respectively. The IL-inseparable residual waste solution was directly used to generate green fluorescent carbon dots (FCDs) by constructing a colloidal solution with the help of a surface-active IL, choline dioctyl sulfosuccinate ([Cho][AOT]). The stability of colloidal FCDs was examined using FTIR, FT-NMR, and Raman spectroscopy. FCDs were extracted from the colloidal solutions *via* the demicellization process and characterized using HR-TEM (2 to 5 nm) and PXRD techniques. The optical properties of colloidal FCDs were measured using UV-Vis and fluorescence spectroscopy and showed a wide range of emission (*λ*_460 nm_ to *λ*_590 nm_). Such FCD stabilized colloidal solutions could be effectively used in fluorescence imaging of yeast cells, thus making the biorefinery approach more sustainable.

## Abstract 2

In this study, a water-miscible ionic liquid (IL), 1-ethyl-3-methylimidazoliumacetate ([EMIM][Ac]), has been used for lipid extraction from marine diatoms *Thalassiosira lundiana* CSIR-CSMCRI 001 by following a non-polar solvent partition method.

## Graphical abstract

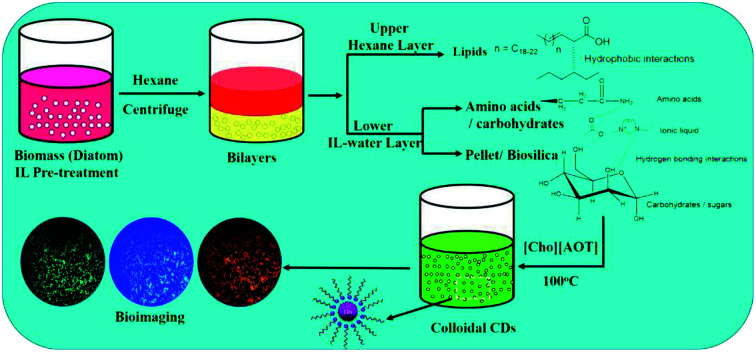

## Introduction

The International Energy Agency (IEA) has reported that over the next five years, renewable energy capacity will be increased and exceed the possible existing energy sources, including natural gas and coal, *etc.* Researchers found that marine biomass such as macroalgae and microalgae are some of the best alternatives towards finding such renewable energy sources. Diatoms, a group of eukaryotic microalgae found in the phytoplankton (Bacillariophyta) of the world's oceans and freshwater, are believed to be responsible for about 1/5 of the primary biomass productivity on earth.^1,2^ Diatoms can fix about 20–25% of atmospheric carbon through photosynthesis in the presence of sunlight.^3^ The shell of the diatoms is made of amorphous hydrated silica called frustules. The biological control of unique frustule morphology (bio-silicification) has attracted the attention of ecologists and materials scientists.^4^ Diatom cells can store large amounts of long-chain saturated fatty acids (LCSFAs), polyunsaturated fatty acids (PUFAs), and fucoxanthin. LCSFAs (C13 : 0–C21 : 0) including palmitic acid, stearic acid, docosanoic acid, arachidic acid, lignoceric acid, myristic acid, nonadecanoic acid, and dodecanedioic acids are the most common energy storage compounds.

Furthermore, PUFAs are long-chain fatty acids that contain more than two double bonds. PUFAs are classified into two prominent families: n-6 and n-3 families derived from linoleic acids (18 : 2) and α-linolenic acids (18 : 3). The most common n-6 PUFAs fatty acids such as linoleic acid (18 : 2), γ-linoleic acid (GLA, 18 : 3), and arachidonic acids (AA, 20 : 4) are involved in membrane synthesis.

n-3 PUFAs such as α-linolenic acid (ALA, 18 : 3), stearidonic acid (SDA, 18 : 4), eicosapentaenoic acid (EPA, 20 : 5), and docosahexaenoic acid (DHA, C22 : 6) are essential fatty acids for higher heterotrophic organisms, such as invertebrates, fish and human. However, heterotrophic organisms do not synthesize n-6 and n-3 PUFA *de nova*.^5^ The importance of long-chain n-3 PUFAs as an anti-inflammatory and in hyperlipidemia by lowering cholesterol and triacylglyceride is well known, and hence these have been reported to reduce cardiovascular systems and risk of atherosclerosis.^6^ LCPUFAs comprise 15–30% of the brain's dry weight; hence, it plays an essential role in brain development.^7^ Lafourcade's research group also reported the nutritional deficiency of n-3 PUFAs linked to neuropsychiatric diseases.^8^ Therefore, due to the high commercial value of LCSFA, PUFA and fucoxanthin biomolecules, an effective and sustainable method is needed for extraction. Many industries are actively engaging in the efficient extraction of fatty acids from microalgae. Biotechnology industries produce bioactive compounds from the diatom biomass used in nutraceutical, pharmaceutical, beauty care products (cosmetics), and biofuels.

There are many advanced experimental techniques such as biochemical, thermochemical, transesterification, and micro-algal fuel cell process for the efficient conversion of bioenergy from microalgae.^9–11^ Actively, many researchers are engaged in developing the microalgae biorefinery technologies to process biomass, including the extraction of lipids, pigments, and other bioactive compounds.^9^ Conventionally, researchers use many toxic inorganics and organic chemicals as solvent media to extract value-added chemicals from microalgae.^12^ Several efforts have been made to develop sustainable and cost-effective extraction processes of lipids and pigments.^12–19^ Previously, Kumar^13^ and Trang^18^ research groups have critically reviewed the advantages and disadvantages of various lipid extraction methods. According to these studies, further research is needed to improve and implement extraction technology.^9–11^ Many biorefinery approaches are utilized conventional volatile organic compounds (VOCs) for the separation/extraction^20,21^ of value-added chemicals from microalgae.^22,23^ Often, uncontrolled emissions of volatile organic compounds (especially solvents) act as greenhouse gases and may cause climate change. In this concern, neoteric solvents of ILs are playing a vital role in the extraction of fatty acids. ILs are a new class of green solvents that replaces conventional volatile organic solvents. Naturally, ILs consist solely of ionic species, which are inorganic anions and organic cations. ILs are mainly unique in negligible vapor pressure at room temperature. These ILs are low melting even below room temperature. The selections of the cation and anion components of the ILs can alter the physical and chemical properties.

Additionally, these solvents are fluidic at room temperature. The advantages of ILs are high quality, reasonable cost, non-flammable, vastly stable at high temperature, and commercially available. Environmentally friendly due to the biodegradability, low toxicity, reproducibility, and biodegradability.^24–26^ ILs are also non-volatile,^27^ low vapor pressure,^19^ high thermo-chemical stability, high electro-conductivity, and remarkable solubilization of various compounds.^28,29^ In the recent past, ILs and ILs mixed solvent systems have been used as a promising alternative to conventional hazardous, toxic, volatile, and highly flammable organic solvents in many chemical or biochemical processes.^15,30,31^ Advanced extraction technologies have been developed using ILs to extract bioactive compounds such as lipids,^31^ carbohydrates,^18^ proteins, vitamins, and other phytochemicals from microalgae.^32^

Orr *et al.* reviewed the latest developments in the use of IL to process microalgae.^33^ Desai and colleagues report that IL, 1-ethyl-3-methylimidazolium dibutyl phosphate (EMIM DBP) is used to pre-treat *H. pluvialis* cells to recover astaxanthin ≥70%.^34^ This article describes an advancement in a sustainable and novel approach to complete diatoms biomass. Here water-miscible IL ([EMIM][Ac]) has been used for total lipid extraction from the diatom *T. lundiana* isolate CSIRCSMCRI 001. Subsequently, the lipid-free IL solution (recovered IL–biomass composite solution; RIBC) containing inseparable residual biomolecules like amino acids, carbohydrates, chlorophyll, and other secondary metabolites were further converted into green fluorescent carbon dots (FCDs). There is significant interest in the evolution of highly fluorescent advanced materials as contrast agents for bioimaging studies.^35^ CDs are the best materials for *in vivo* studies due to their better fluorescence behavior and nontoxic.^26,36,37^ CDs are carbon-based nanostructured materials. Therefore these materials are called carbon dots (CDs/C-Dots). In general, CDs are spherical particles (graphitic fragments) having sizes less than 10 nm. The CDs are exhibiting excellent fluorescence due to having quantized atomic level sizes with many surface properties.^38–40^ By considering the optical properties of the CDs, we employed luminescent CDs loaded colloidal system as a contrast dye for imaging yeast cells.

## Experimental

### Materials and methods

#### Materials

Choline chloride (≥99%), 1-ethyl-3-methylimidazolium acetate [EMIM][Ac] (FT-NMR spectra in Fig. S1 and S2†) and N,O-bis(trimethylsilyl)trifluoroacetamide (BSTFA) were purchased from Sigma Aldrich (Merck). Dioctyl sodium sulfosuccinate (AOT) (>98%), ethylene glycol, methanol, and hexane were purchased from SRL India. Milli-Q water was used wherever required. All the chemicals were of AR grade and used as received.

#### Methods

##### Synthesis of choline dioctyl sulfosuccinate, [Cho][AOT]

**Fig. 1:**
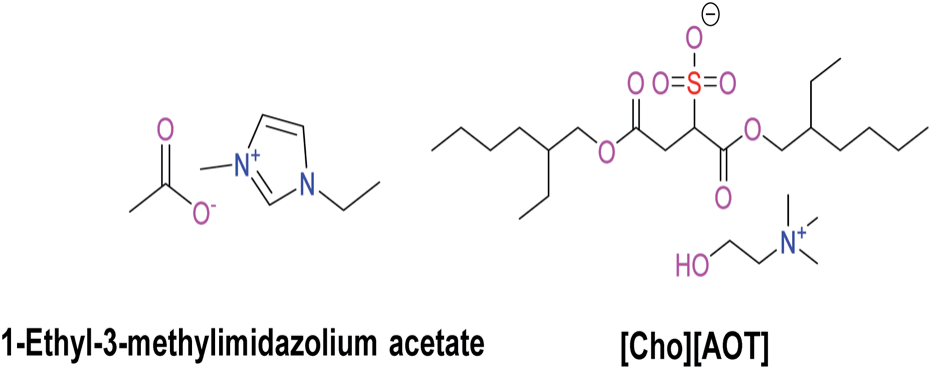
ILs used in this study.

Equimolar amounts of choline chloride and sodium dioctyl sulfosuccinate were dissolved in water and stirred at room temperature for 24 h. Progress of the reaction was monitored using TLC. After completing the reaction, the product was extracted into the DCM layer, followed by washing the DCM layer with a small amount of water several times to make the IL from chloride accessible. Washing was performed until the aqueous layer gives a clear solution even with excess 1 M AgNO_3_. The chloride-free DCM layer was then distilled to obtain pure [Cho][AOT] (Fig. 1) and dried under vacuum for several hours to remove the moisture present and stored in a desiccator. The purity of the [Cho]AOT was ensured from ^1^HNMR, LC-MS techniques (FT-NMR spectra in Fig. S3 and S4†).

^1^H-NMR (600 MHz, DMSO-d_6_) *δ* (ppm) 0.85 (m, 12H), 1.26 (m, 16H), 1.51 (m 2H), 2.80 (m, 2H), 3.10 (m, 4H), 3.41 (s, 9H), 3.66 (m, 1H), 3.90 (m, 5H). ^13^C-NMR (600 MHz, DMSO-d_6_) *δ* (ppm) 171.5, 168.9, 67.4, 66.6, 61.9, 55.7, 53.6, 40.4, 38.6, 34.2, 30, 28.8, 23.6, 22.9, 14.3, 11.2.

ESI-MS: [C_5_H_14_NO]^+^*m*/*z*:104.10 [C_20_H_37_O_7_S]^−^*m*/*z* = 421.2.

##### *T. lundiana* culture and biomass pretreatment

Diatom *Thalassiosira lundiana* CSIRCSMCRI 001 cultures were maintained in modified f/2 media for 15 days in a 5 L culture flask containing 2.5 L of media.^41^ Media was enriched with 0.89 mM NaNO_3_, 29 mM NaH_2_PO_4_ H_2_O, 14.21 mM Na_2_SiO_3_ 9H_2_O, 1.0 ml of f/2 trace elemental solution, 0.5 ml of vitamin solution^42^ under laboratory conditions at 25 ± 1 °C, with an auto programmed light (100 μmol s^−1^ m^−2^) and dark periods (12:12 h L:D). The *T. lundiana* culture's cell number was measured every 3 days using flowCAM-8000 (Fluid Imaging Technology, USA).

##### Total lipid extraction, derivatization, and fatty acid mass profiling

For total lipid extraction, 15 days old diatom cultures were subjected to centrifugation (Kubota 5500) at 8000 rpm for 15 min. After that, the biomass was collected and freeze-dried at −40 °C under vacuum pressure. Then the weighed dry biomass (1.0 g) was resuspended in IL ([EMIM][Ac]) (3.0 g) and subjected to ultra-sonication 33 kHz for 10 min using DTH Sonicator (VWR B9500E). After that, an equal volume of pure hexane was added to the IL–biomass solution and mixed thoroughly, then centrifugated for 15 min at 12 000 rpm for the lipid separation from IL–biomass solution. Further, the hexane solvent is added to the above IL–biomass solution to create a biphasic system that allows for liquid–liquid partitioning to fractionate the mixture. Using a separating funnel, the lipids are collected from the hexane layer. The remaining IL–biomass composite solution (RIBC) was collected and used as a carbon source for CD synthesis protocols. At the same time, the collected hexane fraction was evaporated at room temperature and derivatized with the addition of 100 μl N,O-bis(trimethylsilyl)trifluoroacetamide (BSTFA + 1% TMS, Sigma Aldrich) for non-target fatty acids mass profiling using GC-MS-TQ8030 (Shimadzu Corporation, Kyoto, Japan) in an optimized thermal program.^43^ Molecular mass of each fatty acid were scanned within the range 40–1094 amu using EI scan mode at 70 eV. Finally, the resulted chromatograms were processed using GC-MS Post-run analysis software (Shimadzu), and a similarity NIST 14 library search engine was employed for fatty acids methyl esters (FAME) identifications.

##### Fluorescent carbon dots (CDs) synthesis

For fluorescent CD synthesis, 5 ml of IL-based colloidal solution containing 4.5 ml of RIBC and 0.3 mM of [Cho][AOT] were mixed in a 10 ml reagent vial, heated at 100 °C for 12 h, and then cooled to room temperature.^44,45^ During this, a colorless solution was slowly transformed into a dark brown solution that indicated colloidal CDs.^38,46^ Optical and spectroscopic properties of colloidal CDs were performed using UV-Vis, Fluorescence, FT-IR, FT-NMR, and FT-Raman. For the TEM and PXRD analysis, the CDs loaded micellar (colloidal CDs) solution was subjected to de-micellization using hexane as cosolvent. CDs are collected in hexane layer and centrifuged at 12 000 rpm for 15 min to obtain solid dark brown CDs and washed with water, ethyl acetate, and used for further characterizations.

##### Physicochemical properties of ionic liquid and colloidal solutions

###### Thermogravimetric analysis (TGA)

Thermal stability of ILs ([EMIM][Ac]) and recovered IL solutions (RIL) and fluorescent colloidal CDs solutions were measured over time as a function of temperature using TGA/SDTA851e (Mettler Toledo, US) under a nitrogen atmosphere with gradient temperature ranging from 25 °C to 800 °C, with a heating rate of 5 °C min^−1^.

###### Differential scanning calorimetry (DSC) analysis

Phase transition behavior and thermochemical properties like glass transition temperature (*T*_g_), melting point (*T*_m_), crystallization transition (*T*_c_), and heat capacity of all samples were recorded with 5 °C min^−1^ in −150 to 100 °C range using DSC, 204 F1 Phonex instruments with advanced Proteus software (NETZSCH Proven Excellence, Germany).

###### FT-IR spectrometer

Functional characteristics were evaluated at 25 °C using a remote liquid probe NIR-equipped FT-IR spectrometer (Model, Perkin Elmer, US). Liquid samples such as ILs and colloidal solutions were examined using BaF_2_ windows and a Teflon spacer. In the case of solid samples, KBr pellets were prepared. The optical path length was 0.02 mm. For each spectrum, at least 20 scans were made with a resolution of 0.5 cm^−1^. After that, the material IR spectral signals were processed using Spectrum 10™ software (Perkin Elmer, US).

###### FT-NMR

The chemical changes were also confirmed with FT-NMR Measurements; ^1^H & ^13^C NMR experiments were performed on a JEOL 600 MHz NMR spectrometer.

###### Dynamic light scattering

Dynamic light scattering (DLS) of non-fluorescent and fluorescent solutions was performed with known viscosity and refractive indices of solutions. Measurements were carried out in a quartz cuvette of 1 cm path length at 298.15 K on Spectro Size™ 300 (NaBiTec, Germany) light scattering apparatus with a He–Ne laser at a wavelength of 660–670 nm, and power 100 mW as a source at an angle of 90°. The data evaluation of the DLS measurements was performed with the inbuilt CONTIN algorithm. The temperature of the measurements was controlled with a built-in Peltier device with an accuracy of ±0.1 K.

###### UV-Vis-NIR spectrophotometer

The optical properties of synthesized fluorescent materials were carried out using a UV 3600 Shimadzu UV-Vis-NIR spectrophotometer at 298.15 K. In a typical experiment, the colloidal solutions with synthesized materials (CDs) were taken in a quartz cuvette of 1 cm path length was considered measurements.

###### Fluorescence spectrophotometer

Fluorescence measurements were performed using a Fluorolog (Horiba Jobin Yvon) spectrometer using a quartz cuvette of path length 1 cm. The intrinsic fluorescence spectra of colloidal CD solutions were measured at their respective excitation wavelength. The maximal values of fluorescence are the average of three measurements.

###### Transmission electron microscopy (TEM)

TEM images were taken using a JEOL JEM-2100 electron microscope at a working voltage of 80 kV. Samples were prepared by putting a drop of sample solution on the lacy-coated copper grid (300 mesh) before analysis; it was dried for 24 hours in a vacuum desiccator.

###### Powder X-ray diffraction (PXRD)

X-ray measurements of CDs were performed using XRD, Philips X'pert MPD system with CuKα radiation (*λ* = 1.54056 Å).

###### Fluorescent microscopy bio-imaging study

Active dry yeast powder (Baker yeast) was dissolved in sucrose media for 30 min at 37 °C. After that, a media loop was taken and streaked on Potato Dextrose Agar (PDA) plates and kept at 30 °C for 12 h. After the incubation period, a prominent single colony was transferred into 50 ml fresh liquid media (PD) and kept in an orbital shaking incubator at 30 °C with 60 rmp for 2 days. Two days old, baker yeast cells were stained with fluorescent CDs solution. Similarly, a negative control (without any stain) and positive control (ethidium bromide) stained cells were prepared. Excess stain was removed by washing with phosphate buffer (7.0 pH) and mounted on the microscopic slide. The mounted slides were observed under 40× under bright field followed by fluorescent light with Zeiss filter set 49 equipped fluorescent microscopy (Zeiss AX 10, AFNS Labs, Canada) with autofocus camera and Axio Vision software.

## Results and discussion

### The growth rate of diatoms

Diatom, *T. lundiana* growth, and cell density were exponentially increased up to 9 days. After that, the logarithmic growth was reached the stationary phase due to nutritional depletion in batch cultures. However, the specific growth rate was significantly decreased with increasing the cultivation time (Fig. S5 ESI†). At the end of the stationary phase on the 15^th^ day, 3.4 g l^−1^ of fresh biomass was produced.

### Biomass pretreatment and surface morphology of cells

**Fig. 2:**
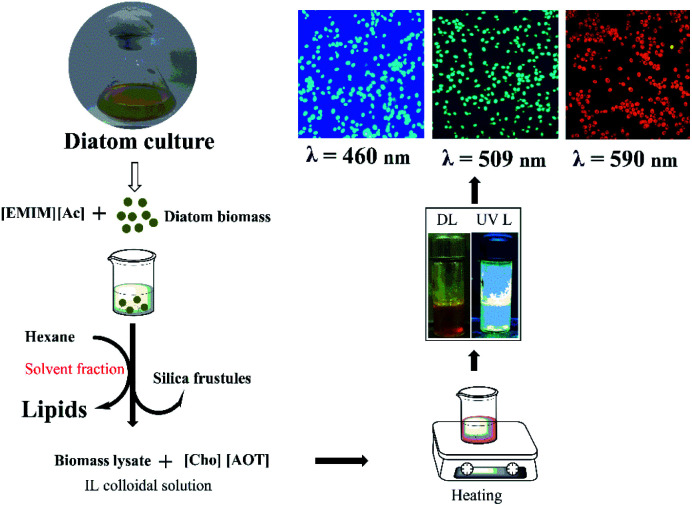
Schematic representations of lipid extraction and hydrothermal method for synthesizing green fluorescent carbon dots (CDs) from biomass residues and its application in bio-imaging technique.

As previously stated, selecting a suitable solvent system is the primary step for extracting and determining the lipid composition from any microalgae. In this study, ILs based solvent system was used to rupture the diatom cell wall, release valuable fatty acids, and then separate by non-polar solvent portioning (Fig. 2).

**Fig. 3:**
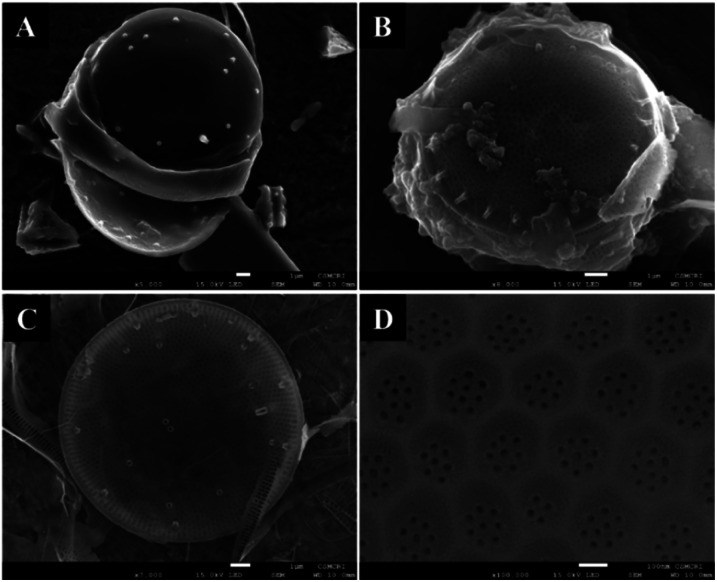
Scanning electron microscopy of centric diatoms *Thalassiosira lundiana* CSIRCSMCRI 001. (A) Untreated frees dried cells, (B) IL treated cells silica frustule (1 μm scale) and (C) recovered silica frustules (biosilica) from IL-water layer and washed with absolute ethanol. (D) The surface topology of the siliceous valves bears in hexagonal cribrums with foramina in 20–22 nm pore size (scale bar – 100 nm).

*T. lundiana* has a unique porous inorganic cell wall, also called silica frustule. Several reports on the morphological and physicochemical properties of diatoms can be used in many energy-related applications.^47–49^ Scanning electron micrographic (SEM) images revealed that the diatom *T. lundiana* isolate was a centric type (Fig. 3).

The morphological features of untreated and IL-treated cells' size, shape, girdle view, and silica surface morphological characteristics are depicted in Fig. 3A and B. Siliceous frustules were centric type and slightly convex in shape. The average size of whole frustules was 12 μm (at 1 μm scale bar).

A tube-like structure called fultoportulae (∼20 nm) was scattered on the surface of the valves. Fig. 3C and D images represent that the recovered mesoporous silica frustules from the IL-water layer. Siliceous valve bearing surfaces in hexagonal cribrums with 7–10 number of foramina (pores). The foraminas were circular with 25 nm in diameter in size.

Extraction of lipids using ILs has shown many advantages in the form of recyclability and efficient extraction. However, there are still a few limitations, such as selectivity, cost-effectiveness, and complete biomass dissolution. Recently, additional choline and imidazolium-based ILs-water mixtures were screened to extract carbohydrates and lipid from two varieties of algae effectively.^18^ In this work, the lipids (30.6% and 51% total lipids) and sugars (71% and 26% total sugars) were extracted using choline amino acid-based ILs. Whereas the IL, 1-ethyl-3-methylimidazolium acetate ([EMIM][Ac]) had shown, the total lipid value somewhat smaller than the conventional methods, which may be due to the lipids still being bounded with lipid droplets or bounded with proteins in the presence of [EMIM][Ac] which are unable to get extracted by hexane without acid neutralization.

### Solvent partitioning

It has been reported that the extraction of total lipids from the biomass such as intact *C. vulgaris*, broken *C. vulgaris*, and *S. platensis* was 2.5%, 4.5%, and 7.2%, respectively, using the [EMIM][Ac] liquor with hexane fraction without acid neutralization.^18^ In another study, the hydrophilic water-miscible IL, 1-butyl-3-methylimidazolium methyl sulfate ([BMIM]MeSO_4_) showed high extraction efficiency of total lipid (21.2%) from algal biomass than hydrophobic IL.^31^ The IL, [TMAm]SO_4_ showed better extraction efficiency of EPA extraction from microalgae.^19^ Kim and co-workers reported that 19.0% of the lipid was extracted from *Chlorella vulgaris* using a mixture of [BMIM][CF_3_SO_3_] and methanol, which was higher than the conventional Bligh and Dyer's method (10.6%).^50^

The previous reports state that ILs are one of the best solvents for lipid and sugar extraction. In this case, we have worked with ILs; ([EMIM][Ac] and [Cho][AOT]), which were used for both lipids and sugars extraction separately. Water-miscible IL, [EMIM][Ac] was used for the dissolution of biomass. After dissolution, the hexane solvent was used to dissolve lipids, create a bilayer system, and phase separation of [EMIM][Ac] treated biomass.

The lipid quantity extracted from *T. lundiana* was 91.4 mg g^−1^ (9.1%). 47 different FAs were detected and identified (Table S1 ESI†). Among these, eleven different fatty acids accounted for more than 1% of total lipid.

**Fig. 4:**
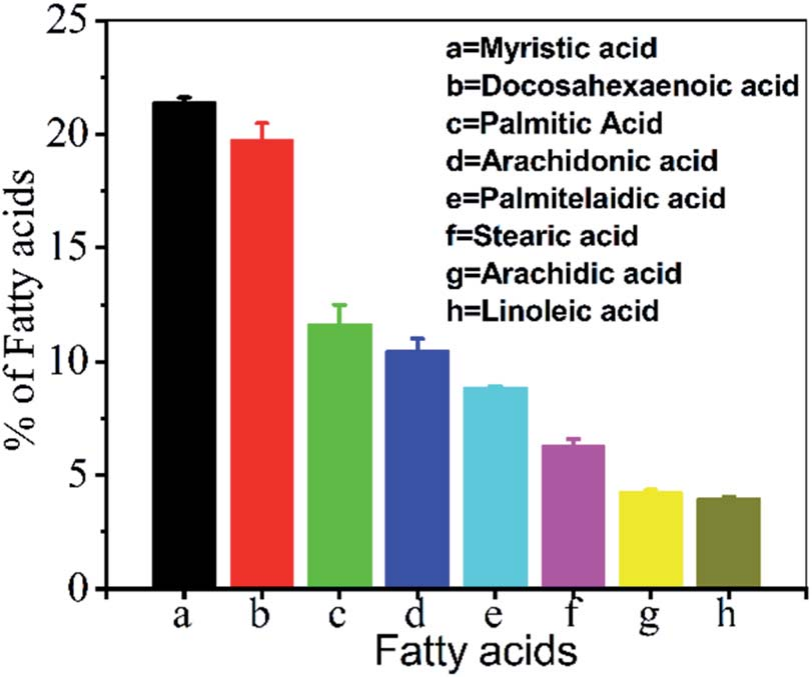
Fatty acids separated from IL pre-treated biomass ([EMIM][Ac]) by non-polar solvent (hexane) partition. Data shown in triplicate mean ± SE, *n* = 3.

Especially, the long-chain saturated fatty acids such as myristic acid (14 : 0, 15.1%), palmitic acid (16 : 0, 11.2%), pentadecanoic acid (15 : 0, 6.1%), stearic acid (18 : 0, 2.4%) and arachidic acid (20 : 0, 3.2%) were dominated. Similarly, monounsaturated fatty acids including 5-dodecenoic acid (12 : 1, 1.2%), oleic acid (18 : 1, n-9, 1.6%), palmitoleic acids (16 : 1 n-7, 8.8%) concentration was higher. Additionally, the essential long-chain unsaturated fatty acids (LCUF) such as linoleic acid (LA, 18 : 2, n-6, 2.9%), arachidonic acid (AA, 20 : 4, n-6, 10.4%), and docosahexaenoic acid (DHA, 22 : 6, n-3, 19.6%) were dominant compounds (Fig. 4). The extraction percentage of LCUF was considerably high from these results, denoting that the selected IL effectively disrupts the diatoms' cell walls. The rest of the biomass residues and other biomolecules remained present in IL-layer.

### Structural stability of [EMIM][Ac] upon biomass treatment

Due to having ionic and polar nature of [EMIM][Ac], the efficacy of [EMIM][Ac] towards biomass dissolution and separation of lipids is well known.

**Fig. 5:**
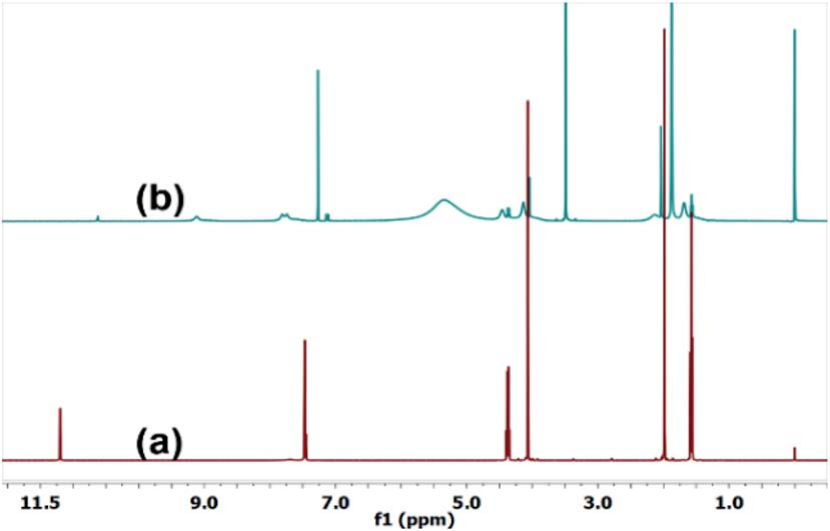
^1^H FT-NMR spectra of (a) pure [EMIM][Ac] and (b) recovered [EMIM][Ac]–water mixture.

Therefore, [EMIM]^+^ cation and [Ac]^−^ anion interacts, having ionic and hydrogen bonding interaction with the rest of the biomass such as sugars, amino acids, and chlorophyll. Generally, ILs are susceptible to decomposition even at elevated temperatures.^51^ TGA analysis reveals the decomposition/degradation temperature of the ILs. Here we performed the analysis of pure IL ([EMIM][Ac]) and RIBC solution (Fig. S6A ESI†). From TGA, the decomposition temperature (*T*_d_) of pure IL ([EMIM][Ac]) at ∼200 °C also appears in the RIBC solution representing the stability of [EMIM][Ac] in the recovered solution. Another transition of recovered IL solution was noticed at 100 °C representing the *T*_d_ of water molecules. Therefore, the TGA profile strongly supports the stability of [EMIM][Ac]. Furthermore, from differential scanning calorimetry (DSC) analysis, the glass transition temperature (*T*_g_) of pure IL, as well as RIBC solution, was analyzed (Fig. S7A ESI†). DSC is one of the critical techniques used to measure the enthalpies of phase transitions, heat capacities, and chemical reactions, *etc.* It offers many significant advantages, such as less sample quantity (milligrams), ease of measurement, versatility, and high-temperature control precision.^52,53^ From DSC, enthalpies can be determined using their *T*_g_ values. In addition, the heat capacities of these ionic liquids have been studied. The *T*_g_ of IL and RIBC solution at ∼−110 °C indicates [EMIM][Ac] structure is hardly affected during the biomass conversion even at elevated temperatures. This is mainly due to its high chemical and thermal stability of IL. The FT-IR and FT-NMR confirmed that the recovered IL is chemically stable during biomass treatment and lipids extraction. From the NMR, the chemical shift values are studied for both pure IL and recover ILs. The presence of pure [EMIM][Ac] and RIBC solutions are recorded (Fig. 5, S1, and S2†). Characteristic chemical shift (*δ*) values, primarily imidazolium protons of [EMIM][Ac] at *f* and *e*, *e*′, (see Fig. S1†) values are 11.2, and 7.5 are present and indicates the stability of ILs after lipid extraction. Similarly, the rest of the ‘*δ*’ values of [EMIM][Ac] appeared in both [EMIM][Ac] and RIBC solutions (see Fig. 5, S1 and S2†). From the FT-IR, the characteristic functional group peaks corresponding to [EMIM][Ac], the *ν*-C=O stretching frequency (str.) due to carbonyl peak of the acetate ion is observed at 1710 cm^−1^ whereas, *ν*-N–H str. of imidazolium ion is observed at 3100 cm^−1^ (Fig. S8 ESI†).

### Synthesis and photophysical properties of CDs

Microalgae biomass is rich in carbon content; therefore, even the extraction of lipids contains carbohydrates, amino acids, and other organic moieties.

**Fig. 6:**
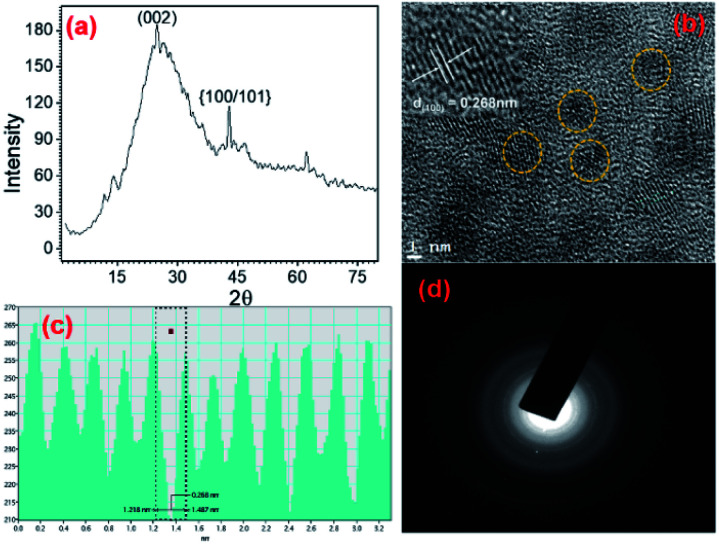
(a) PXRD of pure CDs, (b) HR-TEM images of CDs, (c) *d*-spacing values, and (d) SAED of CDs obtained from HR-TEM.

IL solution, having the above said residual biomass, was further treated with an IL surfactant ([Cho][AOT]) to construct a colloidal formulation (micelles). The micellar structures acted as nanoreactors for the preparation of luminescent materials (CDs) at elevated temperatures (synthesis of CDs are provided in materials and methods). After the synthesis of CDs, the stability of the ILs is examining using FT-NMR studies. The CDs loaded ILs based colloidal system is stable, and there is no change in stability of the ILs (Fig. S10†). CDs were extracted from IL colloidal solutions by cracking the micelles and investigating size, shape, and crystallinity using PXRD and HR-TEM (Fig. 6).

The PXRD peak at ∼23° shows the d(002) and at 43° confirms the *d*{100/101} phase strongly supports the formation of highly crystalline graphitic nature CDs. TEM images showed that as-synthesized CDs have small and narrow size distribution from 2 to 5 nm. The interlayer spacing of CDs shows the *d*-spacing value is *d*(100) 0.268 nm. HR-TEM *d*-spacing values and SAED patterns confirm graphitic carbon nature, and results are comparable to an earlier report.^54^ Conventionally, CDs are synthesized using high carbon content chemicals/bio-polymers at harsh conditions.^39,55^ Here, we have generated the CDs directly from RIBC solution in a bio-friendly and integrated manner. Colloidal CDs solution (CCDs) of RIBC were further examined for their stability and fluorescent properties.

**Fig. 7:**
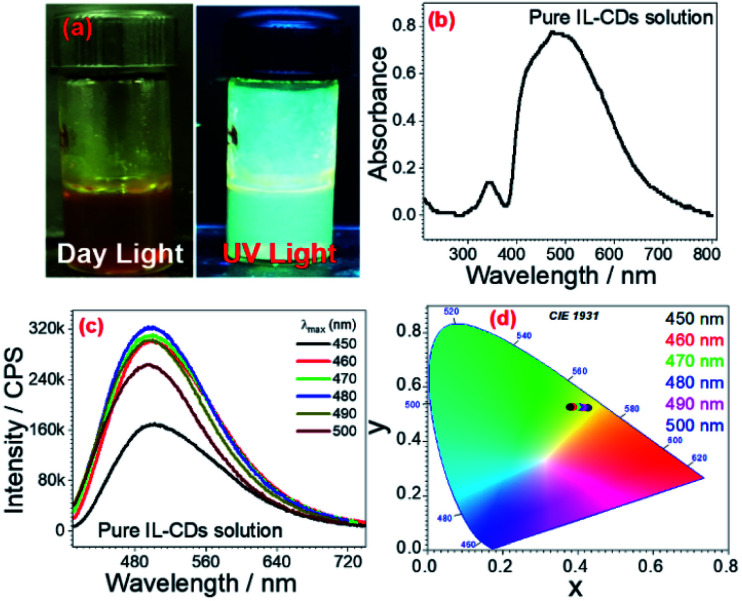
(a) Images represents absence and presence of UV-Vis light (365 nm) pure IL colloidal solution, and CCDs solution, (b) UV spectra, (c) fluoresce emission spectra (d) CIE chromaticity diagram at different excitation energy (for clarity of figure only selected wavelengths such as 450 nm, 460 nm, 470 nm, 480 nm, 490 nm, and 500 nm are provided) of CDs loaded colloidal solution.

UV-Visible spectra of these CCDs were recorded to study the optical properties. CCDs were found to absorb the entire visible region (∼400 nm to ∼700 nm) as shown in (Fig. 7a and b). The emission spectra were recorded at every energy level (from 400 nm to 650 nm) (Fig. 7c and S11†); from the emission spectra, the different excitation wavelengths exhibited their respective emission intensities. At 480 nm, the excitation wavelength is an emission of energy at 495 nm wavelength with the highest intensity indicating bright light emission at this particular wavelength. Other excitation energies (400 nm to 650 nm) also exhibited emission at their respective wavelengths. The excitation wavelengths, from 500 nm to 650 nm, emission gradually decreased. This might be because the colloidal CDs are smaller aggregates and show broad emissions at the entire UV-Vis region. Energy transfer from the outermost electron of CDs from the ground state to the excited state *via* colloidal solution might be happening. Based on the previous report,^56,57^ there must be the trapping of excitons at created surface states of CDs. The passivation of these particular surface states of CDs was done by the surfactant and DES solvent's micelles. Also, it is likely that between two CDs stabilized micelles. There is no difference in surface-related effects.

This allows excitonic energy transfer (EET) among CDs stabilized micelles *via* a dipole–dipole mechanism. The emission of different colors at different excitation values is indicated in the CIE chromaticity diagram. The CIE chromaticity diagram shows that as the energy of wavelength decreases, the emission is shown redshift. CIE coordinates at 500 nm is, *x* = 0.383, *y* = 0.523, 490 nm is, *x* = 0.391, *y* = 0.522, 480 nm is, *x* = 0.397, *y* = 0.521, 470 nm is, *x* = 0.402, *y* = 0.522, 460 nm is, *x* = 0.410, *y* = 0.524, and 450 nm is, *x* = 0.421, *y* = 0.525 (Fig. 7d).

### Bio-imaging studies

**Fig. 8:**
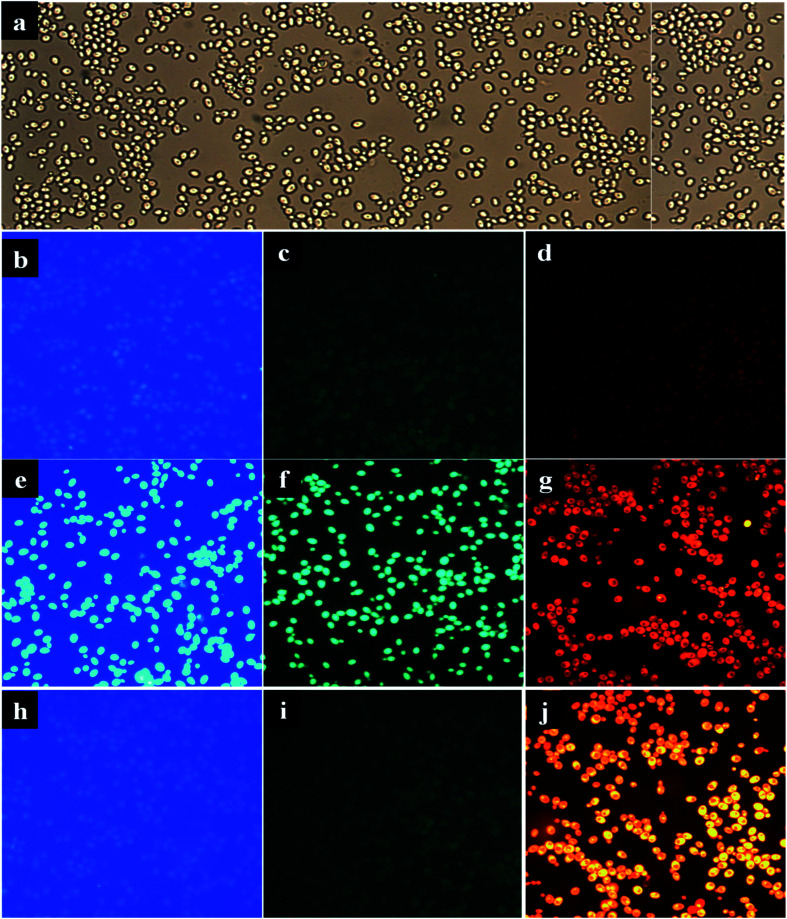
Microscopic imaging of baker yeast cells under different wavelengths. (a) Under bright light, (b–d) control (cells without any stain) under fluorescent light, (e–g) CDs-stained cells under fluorescent light, (h–j) cells stained with EtBr under fluorescent light.

Owing to the high fluorescence intensity of CCDs were investigated for bio-imaging studies. The CDs-stained yeast cells and negative control cells (without any staining), as well as positive control-stained cells (EtBr-stained cells), were imaged under bright light (Fig. 8a) and fluorescent light at different wavelengths (*λ*) (Fig. 8b–d). From the literature survey, we found that there are many types of interactions between DNA and CDs. Milosavljevic *et al.* studied the interaction of DNA with lone pair electrons of oxygen atoms from PEG leads to the formation of hydrogen bonding.^58^

Gao and his co-workers find that the DNA could condense with the amino groups present on the surface of the CQDs.^59^ In a recent study reveals that there is a fluorescence resonance energy transfer (FRET) between photoluminescent CDs and DNA.^60^ Similarly, this study finds that the CCDs having amine groups at the surface might be having interaction with yeast nuclear material *via* hydrogen bonding which results in intense fluorescence bio-images (Fig. 8e–g). The fluorescent emissions were observed from CCDs-stained cells under three different wavelengths *viz.* at 490 nm (green and blue merge), 509 nm (blue), and 590 nm (light orange) regions, whereas positive control stained (EtBr) cells did not show any fluorescence at 490 nm and 509 nm regions (Fig. 8h–j). Similarly, negative control cells also did not show any fluorescence reflectance. Therefore, the synthesized green fluorescent CDs (FCDs) solution can be successfully used as a fluorescence dye for routine DNA or RNA studies with an advantage over the commercially available binding dyes such as EtBr, DAPI, GFP. Commercially available nuclear dyes usually require selective filters, whereas currently synthesized FCDs solution, which exhibits a broad emission spectrum from *λ*_460 nm_ to *λ*_590 nm_, can overcome such a limitation. As a result of the present work highlights the sustainable route for the extraction of lipids as well as green synthesis of CCDs *via* micellar route. The resultant CCDs shows the phenomenal optical properties for bioimaging.

## Conclusions

In this work, the neoteric solvents [Cho][AOT] and [EMIM][Ac] are used for lipid extraction, and CCDs synthesis was effectively performed using marine diatoms biomass. In total, 91.4 mg g^−1^ (dry wt) of lipid was produced from *T. lundiana* CSIRCSMCRI 001 in a single solvent fraction with the help of a classic ILs ([EMIM]Ac) biomass pretreatment. Commercially critical fatty acids such as docosahexaenoic acids (DHA), myristic acid, palmitic acid, and arachidonic acid were 19.6%, 15.1%, 11.2%, and 10.4%, respectively are obtained from extracted lipids. The remaining undissolved biomass lysate–IL solution was directly utilized to synthesize stable fluorescent CD solution by constructing a colloidal solution with the help of a surface-active IL [Cho][AOT]. The colloidal CDs exhibited excellent optical properties with varying light emission (bluish to red) at the different excitation wavelengths (400 nm to 650 nm). The obtained luminescent CCDs are used to visualize the nucleolus in yeast cells under multiple wavelengths *viz. λ*_460 nm_, *λ*_509 nm_, and *λ*_590 nm_. This system is expected to serve as an alternative material for conventional bioimaging applications.

## Conflicts of interest

There are no conflicts to declare.

## Supplementary Material

RA-011-D1RA01425K-s001
