# Supplementary material for: Improved biorefinery pathways of marine diatoms using a water miscible ionic liquid and its colloidal solution: efficient lipid extraction and in situ synthesis of fluorescent carbon dots for bio-imaging applications
Source: RSC Adv. 2021 Jun 22;11(35):21207–15. doi: 10.1039/d1ra01425k (PMC9034194; doi:10.1039/d1ra01425k)
Supplement: RA-011-D1RA01425K-s001 [file RA-011-D1RA01425K-s001.pdf]

Electronic Supplementary Material (ESI) for RSC Advances.
This journal is © The Royal Society of Chemistry 2021

Electronic supplementary information (ESI) for

Improved biorefinery pathways of marine diatoms using a water miscible ionic liquid and its colloidal solution: Efficient lipid extraction and in-situ synthesis of fluorescent carbon dots for bio-imaging applications

Paidi Murali Krishna,[a,b] Veerababu Polisetti,[b] Krishnaiah Damarla,[a,b] Subir Kumar Mandal,[a,b*] and Arvind Kumar[a,b*]*

[a]Academy of Scientific and Innovative Research (AcSIR), Ghaziabad-201002, India

[b]CSIR-Central Salt and Marine Chemicals Research Institute, G. B. Marg,

Bhavnagar-364002, Gujarat, India.

Corresponding authors addresses: Email: Krishnaiah Damarla, damarlakittu@gmail.com; Subir Kumar Mandal, skmandal@csmcri.res.in; and Arvind Kumar arvind@csmcri.res.in

Number of pages: 10

Number of figures: 11

Number of tables: 01

Table of contents

Titles of the figures / tables.	Page No.
**Fig. S1** ^{1}H NMR spectra of pure [EMIM][Ac].	S3
**Fig. S2** ^{13}C NMR spectra of pure [EMIM][Ac].	S3
**Fig. S3** ^{1}H NMR spectra of pure [Cho][AOT].	S4
**Fig. S4** ^{13}C NMR spectra of pure [Cho][AOT].	S4
**Fig. S5** Growth of the diatom *T. Lundiana* CSIRCSMCRI 001 at 25±1°C in f/2 media. (**A**). Logarithmic growth curve, (**B**). Specific growth rate (μ day^{-1}). Data shown in mean ± SE, n=3.	S5
**Table S1** Lipid separated from ionic liquid pre-treated biomass of *Thalassiosira lundiana* CSIRCSMCRI 001. .	S6
**Fig. S6** Thermal gravimetric analysis (TGA) of pure ionic liquid [EMIM][Ac] and recovered ILs after lipid extraction from *T. lundiana* CSIRCSMCRI 001.	S7
**Fig. S7** DSC analysis of pure ionic liquid (EMIMAc) and recovered ILs after lipid fractionation extraction.	S7
**Fig. S8** Fourier transform infrared spectroscopy annotation of pure ionic liquid [EMIM][Ac] and recovered ILs after the lipid extraction.	S8
**Fig. S9** Fourier transform infrared spectroscopy annotation of pure colloidal solution ([EMIM][Ac]+ AOT) and CDs loaded fluorescent colloidal solution.	S8
**Fig. S10** FT-NMR spectral confirmation of compositional changes in synthetic colloidal solution during fluorescent carbon dots synthesise. (**A**) Pure synthetic colloidal solution ([EMIM][Ac]+AOT), (**B**) Fluorescent carbon dots synthesised colloidal solution.	S9
**Fig. S11** Fluoresce emission spectra at different excitation energy (400nm to 650nm, are provided) of CDs loaded colloidal solution.	S10

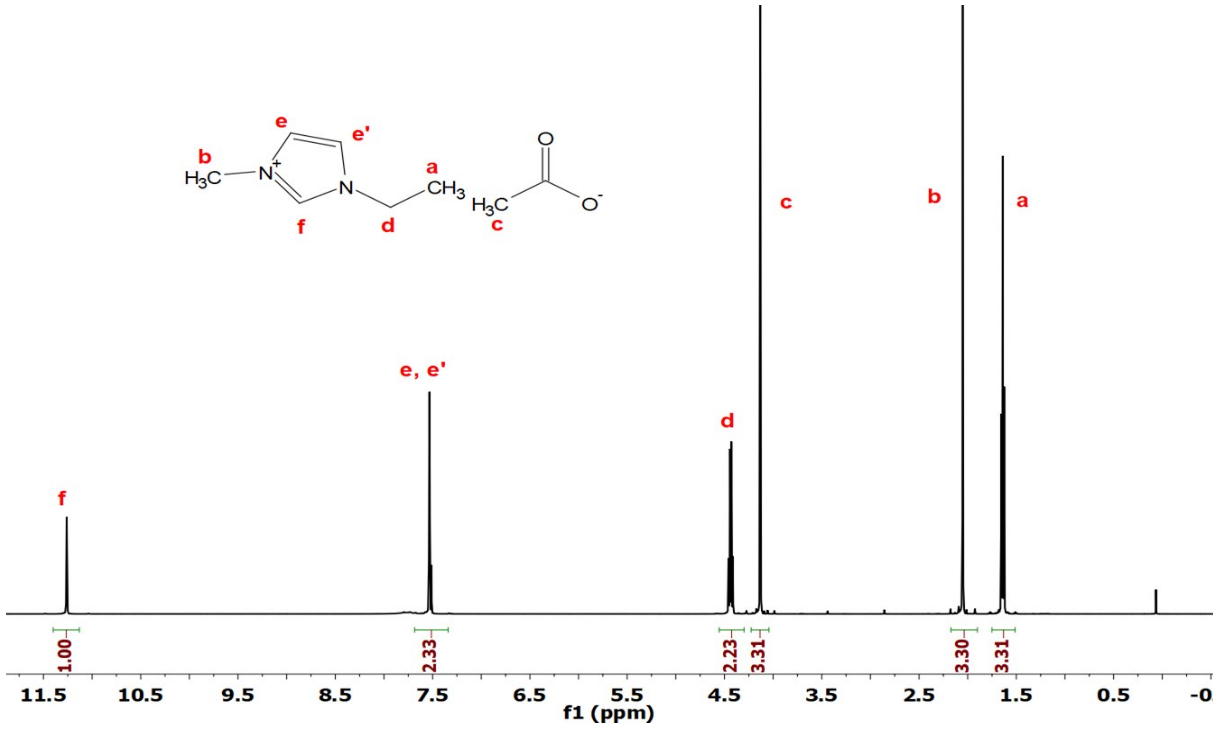

Fig. S1 ^{1}H NMR spectra of pure [EMIM][Ac].

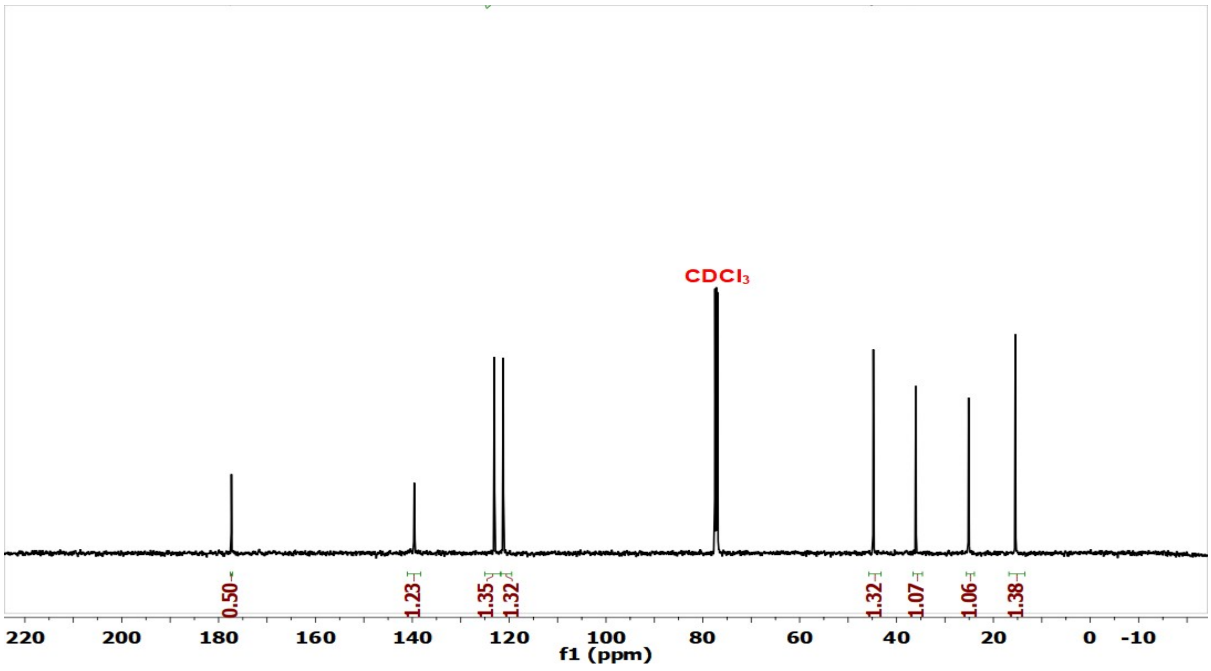

Fig. S2 ^{13}C NMR spectra of pure [EMIM][Ac].

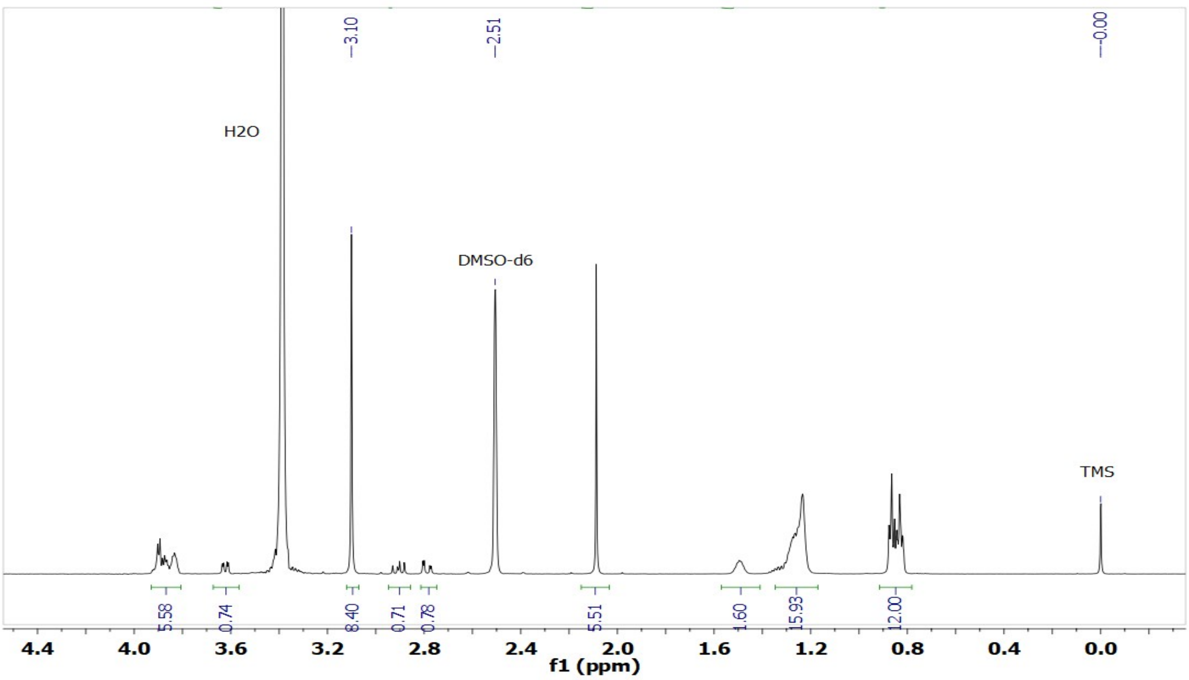

Fig. S3 ^{1}H NMR spectra of pure [Cho][AOT].

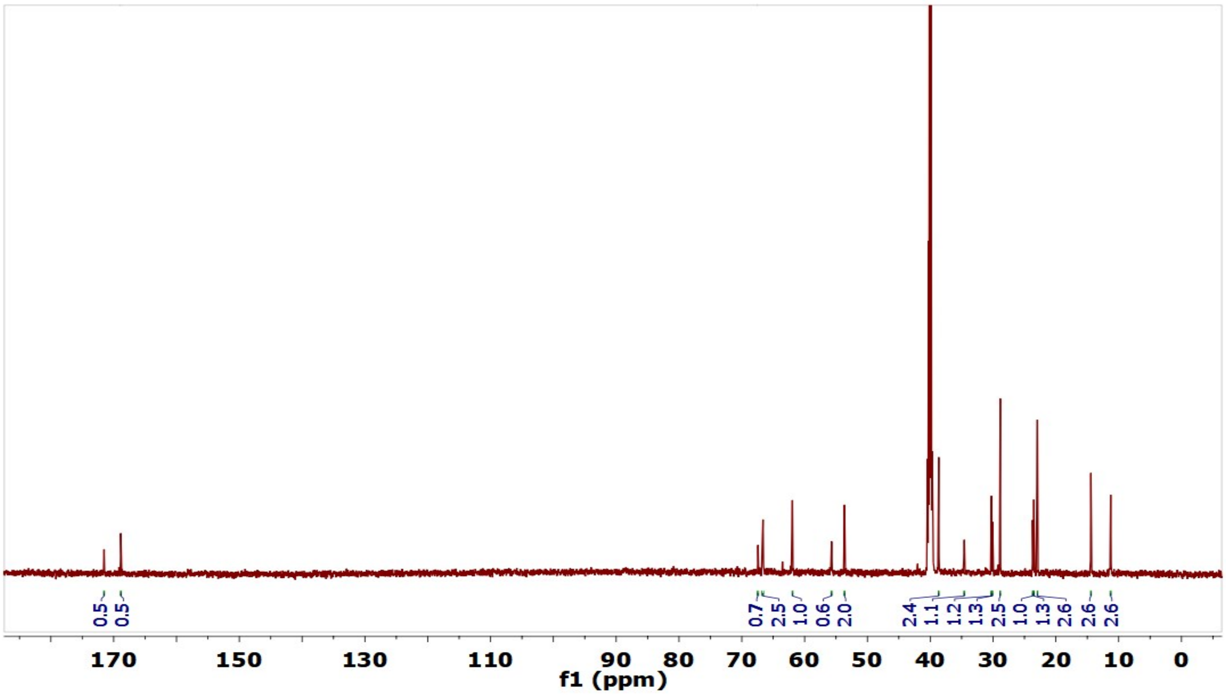

Fig. S4 ^{13}C NMR spectra of pure [Cho][AOT].

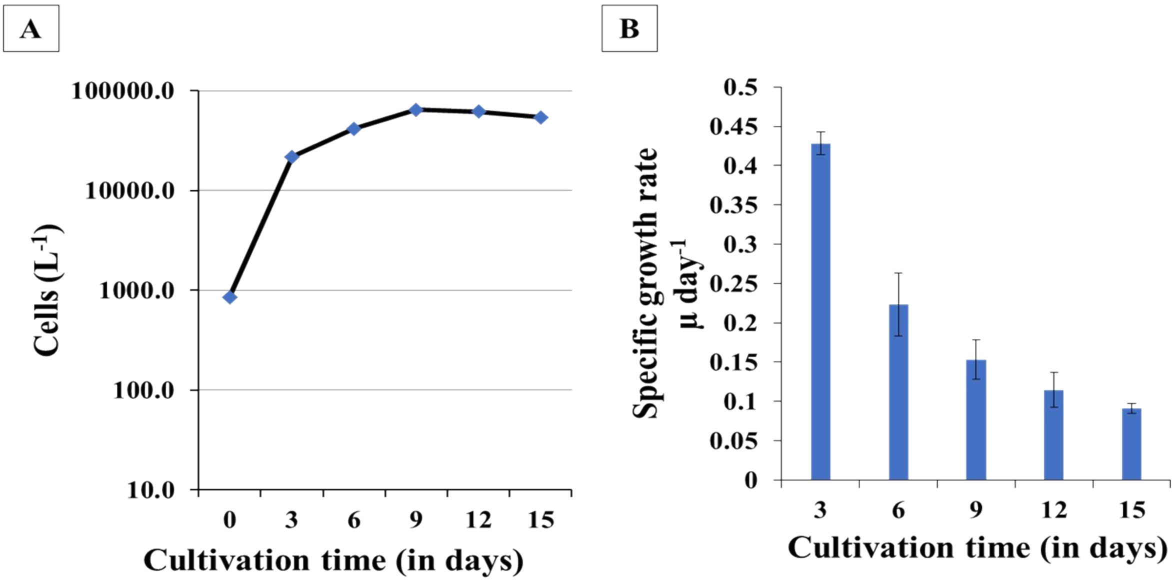

Fig. S5 Growth of the diatom *T. Lundiana* CSIRCSMCRI 001 at 25±1°C in f/2 media. (A). Logarithmic growth curve, (B). Specific growth rate (µ day^{-1}). Data shown in mean ± SE, n=3.

Table S1. Lipid separated from ionic liquid pre-treated biomass of *Thalassiosira lundiana* CSIRCSMCRI 001.

S.No.	Fatty acid composition		% of FAs in total lipid
1	C8:0	Octanoic acid, TMS	0.17
2	C8:1	3-Octenoic acid, TMS	2.40
3	C10:0	Decanoic acid, TMS	1.46
4	C12:0	Dodecanoic acid, 4,6-dimethyl-	1.87
5	C12:1	5-Dodecenoic acid, TMS derivative	1.20
6	C14:0	Myristic acid, TMS derivative	21.40
7	C14:1	9-Tetradecenoic acid, TMS derivative	0.22
8	C16:0	Palmitic Acid, TMS derivative	11.63
9	C16:1, n-7	Palmitelaidic acid, TMS derivative	8.84
10	C18:0	Stearic acid, TMS derivative	6.28
11	C18:1, n-9	Oleic Acid, TMS derivative	1.65
12	C18:2, n-6	Linoleic acid, 3TMS derivative	3.98
13	C19:0	Nonadecanoic acid TMS derivative	0.22
14	C19:3	Z,Z,Z-4,6,9-Nonadecatriene	0.20
15	C20:4, n-6	Arachidonic acid, TMS derivative	10.48
16	C20:0	Arachidic acid, TMS derivative	4.25
17	C22:6, n-3	Docosahexaenoic acid, TMS derivative	19.74
18	C26:0	Hexacosanoic acid, TMS derivative	0.57
19	C27:2	Desmosterol, TMS derivative	2.30
20	C28:0	Octacosanoic acid, TMS derivative	0.85
21	C30:0	Triacontanoic acid, TMS derivative	0.29
		Total	100 %
		Total fatty acids (mg/g dry weight)	90.56
		% of total lipids	9.06

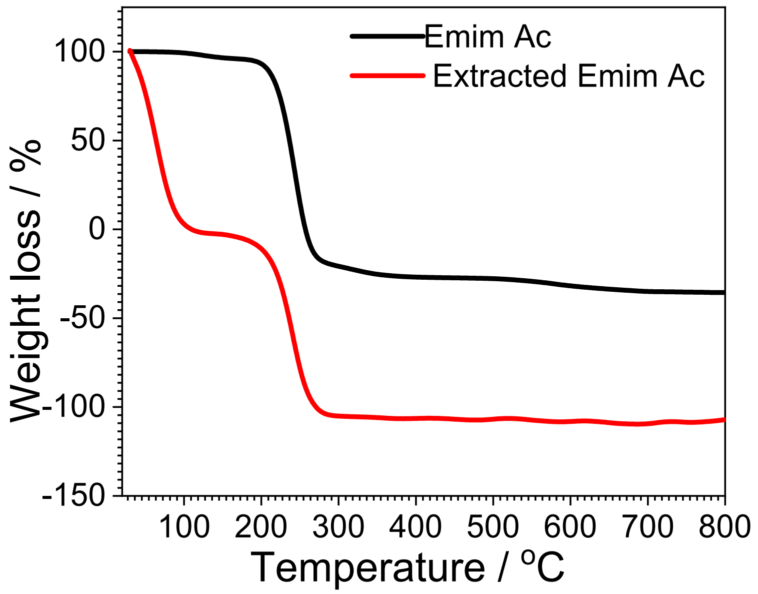

Fig. S6 Thermal gravimetric analysis (TGA) of pure ionic liquid [EMIM][Ac] and RIBC solutions after lipid extraction from *T. lundiana* CSIRCSMCRI 001.

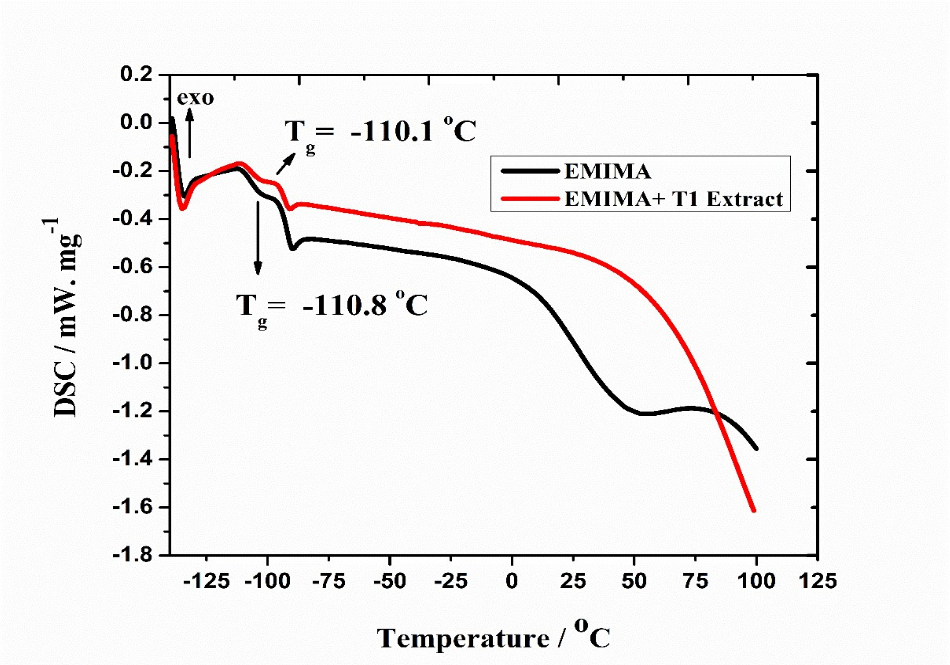

Fig. S7 DSC analysis of pure ionic liquid (EMIMAc) and RIBC solutions after lipid fractionation extraction.

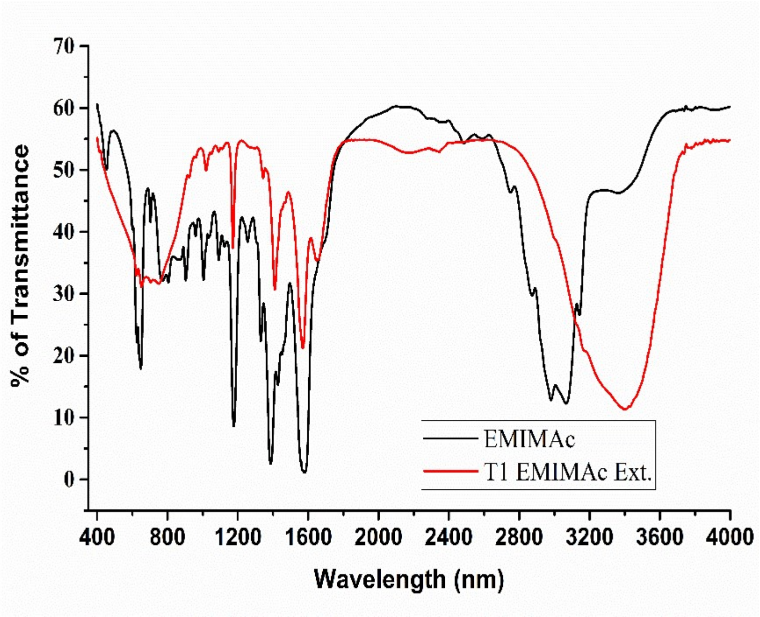

Fig. S8 Fourier transform infrared spectroscopy annotation of pure ionic liquid [EMIM][Ac] and RIBC solutions after the lipid extraction.

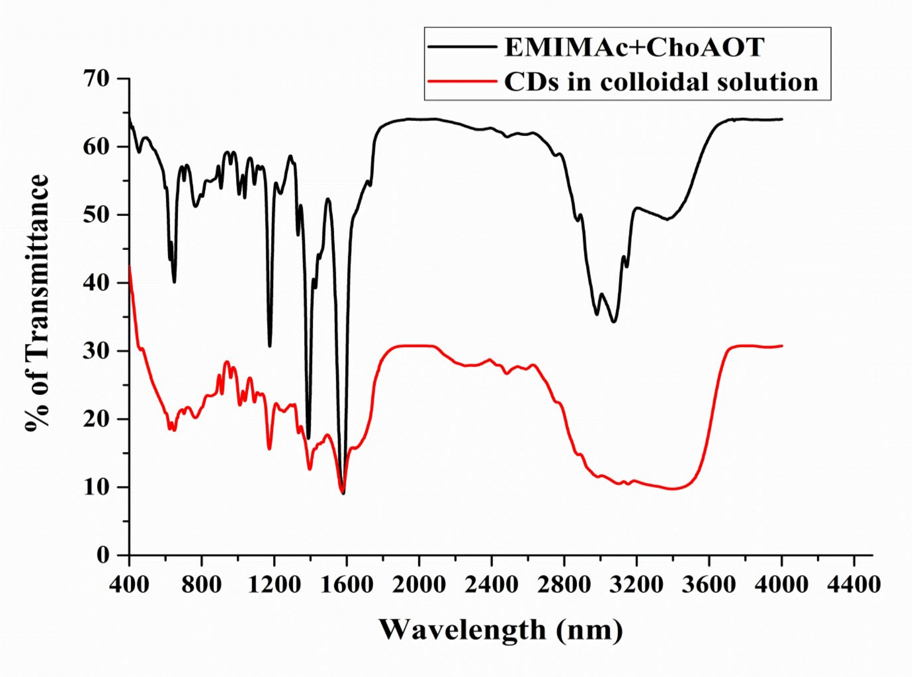

Fig. S9 Fourier transform infrared spectroscopy annotation of pure colloidal solution ([EMIM][Ac]+ AOT) and CDs loaded fluorescent colloidal solution.

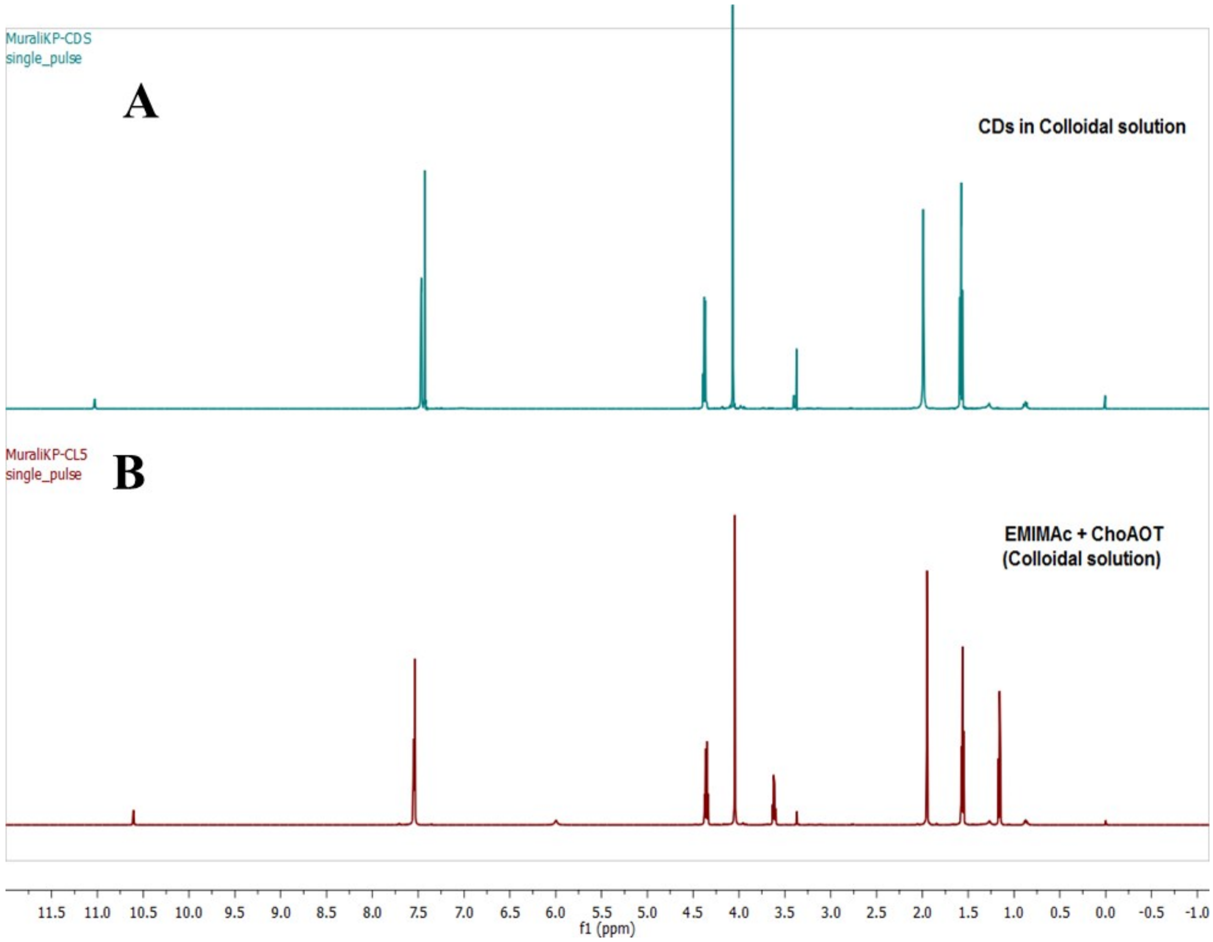

Fig. S10 FT-NMR spectral confirmation of compositional changes in synthetic colloidal solution during fluorescent carbon dots synthesise. (**A**) Pure synthetic colloidal solution ([EMIM][Ac]+AOT), (**B**) Fluorescent carbon dots synthesised colloidal solution.

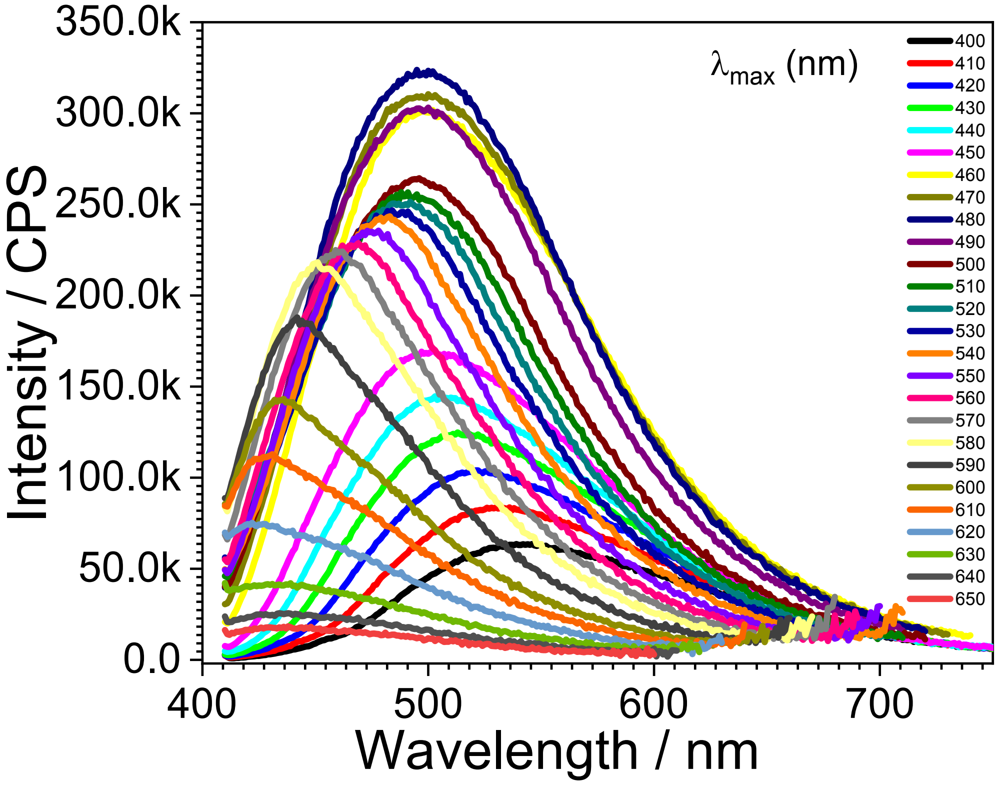

Fig. S11 Fluoresce emission spectra at different excitation energy (400nm to 650nm, are provided) of CDs loaded colloidal solution.
